# Modulating Role of Co-Solutes in Complexation between Bovine Serum Albumin and Sodium Polystyrene Sulfonate

**DOI:** 10.3390/polym14061245

**Published:** 2022-03-19

**Authors:** Matjaž Simončič, Miha Lukšič

**Affiliations:** Faculty of Chemistry and Chemical Technology, University of Ljubljana, Večna pot 113, SI-1000 Ljubljana, Slovenia; matjaz.simoncic@fkkt.uni-lj.si

**Keywords:** protein-PE complexation, solid-liquid phase separation, sucrose, sucralose, molecular crowding, electrostatic screening

## Abstract

The action of three types of co-solutes: (i) salts (NaCl, NaBr, NaI), (ii) polymer (polyethylene glycol; PEG-400, PEG-3000, PEG-20000), and (iii) sugars (sucrose, sucralose) on the complexation between bovine serum albumin (BSA) and sodium polystyrene sulfonate (NaPSS) was studied. Three critical *p*H parameters were extracted from the *p*H dependence of the solution’s turbidity: $pH_{c}$ corresponding to the formation of the soluble complexes, $pH_{\Phi }$ corresponding to the formation of the insoluble complexes, and $pH_{\mathrm{opt}}$ corresponding to the charge neutralization of the complexes. In the presence of salts, the formation of soluble and insoluble complexes as well as the charge neutralization of complexes was hindered, which is a consequence of the electrostatic screening of attractive interactions between BSA and NaPSS. Distinct anion-specific trends were observed in which the stabilizing effect of the salt increased in the order: NaCl < NaBr < NaI. The presence of PEG, regardless of its molecular weight, showed no measurable effect on the formation of soluble complexes. PEG-400 and PEG-3000 showed no effect on the formation of insoluble complexes, but PEG-20000 in high concentrations promoted their formation due to the molecular crowding effect. The presence of sugar molecules had little effect on BSA-NaPSS complexation. Sucralose showed a minor stabilizing effect with respect to the onset of complex formation, which was due to its propensity to the protein surface. This was confirmed by the fluorescence quenching assay (Stern-Volmer relationship) and all-atom MD simulations. This study highlights that when evaluating the modulatory effect of co-solutes on protein-polyelectrolyte interactions, (co-solute)-protein interactions and their subsequent impact on protein aggregation must also be considered.

## 1. Introduction

The interaction between a protein and a polyelectrolyte (PE), which are usually but not necessarily oppositely charged at a given *p*H of the solution, can lead to the formation of protein-PE complexes. The association between the complexes can further lead to two types of phase separation: liquid-liquid (coacervation) or solid-liquid (precipitation), though discriminating between them can sometimes be quite challenging. In general, PEs with a low charge density (so-called “weak” or “annealed” PEs) tend to form coacervates, while strongly interacting polyelectrolytes (so-called “strong” or “quenched” PEs) whose degree of ionization is *p*H-independent, form precipitates [1]. In both cases the previously homogeneous solution separates into two immisible phases: a macromolecule rich phase and a macromolecule poor phase. Such phase separation usually depends on several factors [2]: (i) structural parameters such as charge stoichiometry, charge density (charge patchiness of the protein surface), molecular weight of the components, etc. (ii) preparation parameters (method of solution preparation and the order of mixing of the components, the mixing ratio, kinetics of complexation, etc.), and (iii) media parameters such as the concentrations of the components, *p*H and ionic strength of the medium, the presence of co-solutes, etc.

Protein-PE complexes are being implemented in various industrial and biotechnological applications ranging from protein purification [3,4,5,6], enzyme activation [7], drug delivery [8,9,10], biosensors [11], stabilization in food emulsions [12] etc. Protein-PE interactions are also important in in vivo processes such as the formation of membraneless organelles [5,13] through liquid-liquid phase separation or the interaction between natural polyelectrolytes (DNA) and proteins (histones) [14]. As protein-PE complexation falls under the domain of PE complexation in general, it has been thoroughly investigated: for reviews on PE complexation see refs. [2,15,16,17], for a review about protein-PE complexation see refs. [18,19,20].

The major driving force of protein-PE complexation are electrostatic interactions between charged macromolecules [21,22,23], which can be either repulsive or attractive depending mainly on the nature of the components and the *p*H of the solution. The latter greatly impacts the charge distribution on the protein surface or in case of weak PEs the charge density of the polyions. The formation of complexes is accompanied by a decrease in the free energy of the system which can be broken down into an enthalpic component, caused by the Coulombic attraction between opposite charges, and an entropic component due to the release of counterions upon association [22,23]. Even though electrostatic interactions are the driving force of protein-PE complexation, hydrogen bonding [24] and the hydrophobic effect [25] can also play a role.

**Figure 1:**
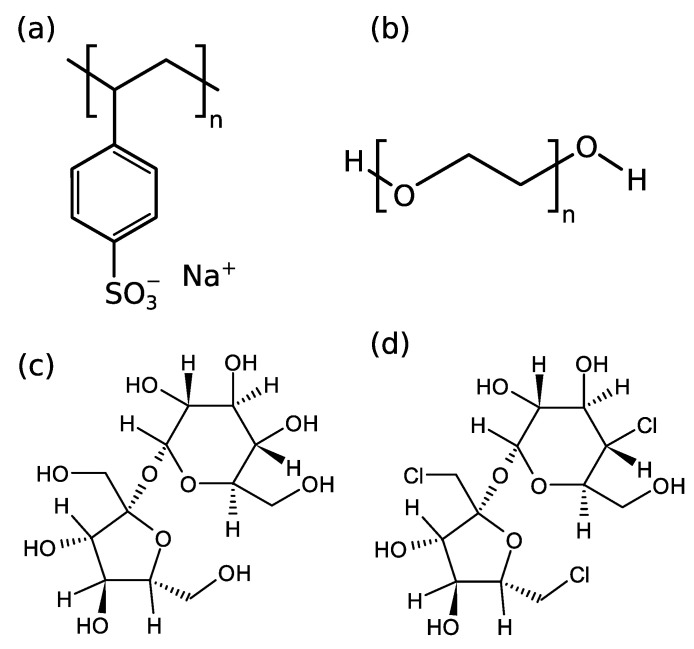
Chemical structures of (**a**) repeating unit of sodium polystyrene sulfonate (NaPSS), (**b**) repeating unit of polyethylene glycol (PEG), (**c**) sucrose, and (**d**) sucralose.

In the present study we focused on the influence of various co-solutes on the complexation between a globular protein, bovine serum albumin (BSA), and a synthetic polyelectrolyte, sodium polystyrene sulfonate (NaPSS; cf. Figure 1a). Although a protein can also be considered a polyelectrolyte (polyampholite), we will use the term “polyelectrolyte” in the context of this article only in reference to NaPSS. BSA has already been investigated in regard to its interaction with cationic PEs, such as PDMDAAC [26], PDADMAC [27,28,29], PMAPTAC [27,30], chitosan [31], as well as anionic PEs, e.g., heparin [32], sugar beet pectin [33], poly(aspartic acid) [34], PAMPS [27] or NaPSS [26,35,36]. However, the modulation of BSA-NaPSS interactions by co-solutes has, at least to our knowledge, not been so systematically addressed. We have studied the effect of three main types of co-solutes: (i) salts (NaCl, NaBr, NaI), (ii) polyethylene glycol (PEG; cf. Figure 1b) with different molecular weights (400, 3000, and 20,000 g/mol), and (iii) sugars (sucrose, sucralose; cf. Figure 1c,d).

Low molecular weight salts added to the solution containing charged macromolecules usually screen the electrostatic interactions at lower to medium concentrations (ionic strengths) but enhance the complexation at higher concentrations owing to the secondary aggregation of protein-PE complexes, which leads to macroscopic flocculation [17,29,37]. The effect also depends on the chemical identity of the added salt [38]. PEG is a well known precipitation agent for protein solutions [39,40] and usually plays the role of the molecular crowder. The assumption in most macromolecular systems is that PEG does not interact directly with proteins, however some studies hint at weak direct interactions between hydrophobic pockets of the protein surface and high molecular weight (MW) PEGs at high concentrations [41,42]. Sugars such as trehalose, glucose, and sucrose are well known for their biopreservative properties, which is a consequence of their water structuring capability around biomacromolecules [43,44]. The cause is centered around the premise that sugars are preferentially excluded form the protein surface, a mechanism which was recently also proposed in regard to protein-PE complexation in the presence of sugars [45].

In this paper we evaluate the modulative effect of aforementioned co-solutes on the phase separation in the BSA/NaPSS system and present mechanistic explanations for the effects.

## 2. Materials and Methods

### 2.1. Materials

Bovine serum albumine (fatty acid-free; LOT number: SLCB1005), hen egg-white lysozyme (LOT number: K49054981), sodium polystyrene sulfonate (average molecular weight 70,000 g/mol), polyethylene glycols with an average molecular weight of 400 g/mol (PEG-400) and 3000 g/mol (PEG-3000), sucrose, sucralose, and NaI (>99%) were purchased from Sigma Aldrich, Burlington, MA, USA. Alkaline metal salts of >99% purity (NaCl, NaBr), concentrated acetic acid (CH_3_COOH), sodium acetate (CH_3_COONa), and 1 mol/L NaOH solution were purchased from Merck KGaA. Polyethylene glycol with an average molecular weight of 20,000 g/mol (PEG-20000) was obtained from Fluka.

### 2.2. Preparation of Buffer, Protein and Polyelectrolyte Stock Solutions

The *p*H was measured using the Iskra *p*H meter (Iskra, Horjul, Slovenia) and a combined glass micro-electrode InLab Micro (Mettler Toledo, Greifensee, Switzerland). Milli-Q water was used to prepare all the solutions.

Acetate buffer was prepared by dissolving the appropriate amount of sodium acetate in water, adding the corresponding amount of concentrated acetic acid and adjusting the *p*H to the desired value by adding small amount of 1 M (mol/L) sodium hydroxide solution. Three buffers with an ionic strength of 0.1 M were prepared: acetate buffer with *p*H = 4.2 (total buffer concentration being 0.46 M), *p*H = 4.6 (total buffer concentration being 0.20 M) and *p*H = 5.8 (total buffer concentration being 0.11 M). A high ionic strength buffer (I=0.1 M) was used to maintain a relatively constant *p*H (±0.1) value of the solution even with the addition of co-solutes. Furthermore, the addition of HCl to a low ionic strength BSA-NaPSS solution resulted in abrupt complex formation, which prevented experimental determination of the turbidity curve in the *p*H range studied.

NaPSS was first purified by dialysis of the aqueous solution using the Dialysis tubing cellulose membrane (Sigma Aldrich, Burlington, Burlington, MA, USA; MW cut-off: 14,000 g/mol) against Milli-Q water until the conductivity of the dyalizate matched that of water (less than 2 μS/cm). The solution was then liofilized using the HETOS-ICC freeze dryer (CD 52-1) and the dry NaPSS was stored for further use.

The stock solutions of proteins (BSA, lysozyme) and NaPSS were prepared in the acetate buffer. Protein powder was dissolved in the buffer and the solution was dyalized against the corresponding buffer for 24 h using the Spectra/Por membrane (MW cut-off: 3500 g/mol). The buffer solution was replaced every 8 h. NaPSS stock solutions were prepared by dissolving the liofilized powder in the same acetate buffer as the proteins. All stock solutions were filtered through 0.45 μm filter (Minisart Sartorius). The concentration of the protein in the stock solution was determined using the NanoDrop OneC Microvolume UV-Vis Spectrophotometer (Thermo Scientific, Pittsburgh, Pennsylvania) at 280 nm (ε = 0.665 L g${}^{-1}$ cm${}^{-1}$ and 2.635 L g${}^{-1}$ cm${}^{-1}$ at 25 ${}^{{}^{\circ}}C$ for BSA [46] and lysozyme [47], respectively). The concentration of NaPSS was determined using a Cary 100 Bio (Varian, Palo Alto, CA, USA) spectrophotometer at 262 nm (ε = 1.82 L g${}^{-1}$ cm${}^{-1}$ at 25 ${}^{{}^{\circ}}C$ [48]). In this work, the concentration of NaPSS is given as the concentration of monomoles per liter of solution. The NaPSS to BSA concentration ratio was defined as $r={\left[\mathrm{NaPSS}\right]}_{\mathrm{monomol}}/\left[\mathrm{BSA}\right]$, where ${\left[\mathrm{NaPSS}\right]}_{\mathrm{monomol}}$ denotes the monomolar concentration of NaPSS, and [BSA] denotes the molar concentration of BSA.

The preparation of working solutions is described in the following sections.

### 2.3. Turbidimetric Titrations

UV-Vis measurements were performed using a Cary 100 Bio spectrophotometer (Varian, Palo Alto, CA, USA) with a Peltier block for temperature regulation, along with a Cary temperature controller (Agilent, Santa Clara, CA, USA) pre-thermostat. Turbidity measurements were recorded at 420 nm in quartz cuvettes with 1 cm optical path length at 25 ${}^{{}^{\circ}}C$. The turbidity, τ, is defined as:

(1) $$\tau =-\ln(I/I_{0})$$

where *I* is the intensity of the light beam after passing through the sample and $I_{0}$ is the incident light beam intensity. None of the macromolecules used (BSA, NaPSS) in the solution absorbs light at 420 nm. The changes in turbidity can therefore be related to the extent of BSA-PE complex formation.

Initial BSA/NaPSS solutions (with or without added co-solutes) were prepared from the BSA and NaPSS stock solutions in acetate buffer (*p*H = 5.8, I=0.1 M). Appropriate amounts of the stock solutions were mixed in a glass flask to obtain a solution with a BSA concentration of 100 μM and a NaPSS concentration of 4000 μM. The ratio of the concentrations of NaPSS and BSA was $r={\left[\mathrm{NaPSS}\right]}_{\mathrm{monomol}}/\left[\mathrm{BSA}\right]=40$. The solution was filtered through a 0.45 μm filter (Minisart Sartorius) and transferred to quartz cuvettes. The turbidity of filtered and unfiltered solutions was measured, however no differences were observed, indicating that filtering did not remove any formed complexes which could be detected by the spectrometer. The *p*H was gradually decreased by adding 10 μL aliquots of 0.2 M HCl and the solution was well stirred after each addition of the acid. The measured turbidities are given as a function of the solution’s *p*H. Considering that NaPSS is a strong PE, which means that its overall charge is not affected by changes in *p*H of the solution, the formation of BSA-PE complexes (increase in turbidity) is related to the changes in the net charge of the BSA and the charge distribution on the BSA surface.

In a few cases, an optical microscope (Olympus CK30/CK40, Japan) was used to capture images of the BSA-PE precipitates formed.

### 2.4. Fluorimetry

Fluorimetric measurements were performed using a LS 55 Perkin Elmer (USA) fluorimeter at 25 ${}^{{}^{\circ}}C$, with temperature controlled by a Perkin Elmer PTP-1 Peltier system. The excitation wavelength was set at 280 nm and the emission scan range was from 300 to 450 nm. The bandwidth for the excitation and emission slits was 5 nm. The emission spectrum of 0.5 μM BSA was measured in the presence of different co-solutes (PEG-3000, PEG-20000, sucrose and sucralose) at different co-solute concentrations. The emission spectrum of 2 μM lysozyme was measured only in the presence of the sugars. Solutions were prepared in acetate buffer with an ionic strength of 0.1 M and *p*H values of 4.2 and 5.8 for BSA and *p*H = 4.6 for lysozyme. Spectra were measured immediately after preparing the solutions. The fluorescent property of a protein is attributed to its hydrophobic amino acid residues, which is why molecular interactions with those residues lead to a change (decrease) in fluorescence intensity. The phenomenon of fluorescence quenching is usually described by the following Stern-Volmer equation [49]:

(2) $$F_{0}/F=1+K_{\mathrm{SV}}\left[Q\right]$$

where $F_{0}$ and *F* represent the fluorescence intensities (recorded at 347 nm) in the absence and presence of the quencher, respectively, $K_{\mathrm{SV}}$ is the Stern-Volmer constant and [*Q*] is the molar concentration of the quencher (added co-solute in our case). By repeating the experiments 3 times, we estimated the uncertainty for the data points to be ±0.1 (the uncertainty was calculated as the largest difference of a single measurement from the mean). The uncertainty in $K_{\mathrm{SV}}$ was determined from the least-squares fit (Levenberg–Marquardt algorithm).

### 2.5. Circular Dichroism (CD)

CD measurements were performed to verify whether the addition of sugar (sucrose/sucralose) had an effect on the secondary structure of the protein. The mean residue ellipticity, [θ], was measured at 222 nm for solutions containing 4.5 μM BSA in the presence of sugar concentrations (up to 400 mM). Acetate buffer was used as the solvent (*p*H = 4.2, *I* = 0.1 M). Measurements were performed with a Jasco-1500 CD spectrometer using quartz cuvettes (optic path length of 0.1 cm) at 25 ${}^{{}^{\circ}}C$. The temperature was controlled with a Julabo F25-ME thermostat.

## 3. Theoretical Methods

To supplement experimental findings, we performed 25 ns all-atom molecular dynamics (MD) simulations of bovine serum albumin in water with added sucrose or sucralose molecules. The simulations were carried out using the GROMACS 2018.1 molecular dynamics software [50] with the all-atom GROMOS 54A7 [51] force field and the SPC/E water model [52]. The simulations were similar to those performed in our previous work on lysozyme-sugar solutions [53] with only minor differences in terms of the number of molecules and the size of the simulation box (L≈15 nm). The starting conformation of the protein was taken from the Protein Data Bank (PDB ID: 4f5s [54]). The box contained one BSA molecule, approximately 9.8 × 10${}^{4}$ water molecules, and 508 sugar molecules, corresponding to a 0.25 M solution of the sugar (a dilute solution of ∼10 wt%).

The simulations were performed for two *p*H values below and above the isoionic point of BSA. By setting the net charge of the protein to +44*e* and −11*e*, as determined by the PROPKA software [55], we were able to mimic the surface charge distribution for *p*H = 4.2 and *p*H = 5.8, respectively (the charge was balanced with a corresponding number of chlorine atoms). The charges correspond roughly to the experimentally determined net charges of BSA as tested by potentiometric titration at the ionic strength of 0.1 M [56], which was in our case the ionic strength of the buffer. To ensure the isotropic arrangement of solvent and sugar molecules around the protein we performed the energy minimization of the initial systems according to the process described in Ref. [53]. A 25 ns simulation of all constructed systems was performed in the isobaric-isothermal (N,P,T) ensamble and statistics was collected on this production run.

The analysis focused on the distribution of sugar and water molecules around the protein surface for both *p*H values.

## 4. Results and Discussion

**Figure 2:**
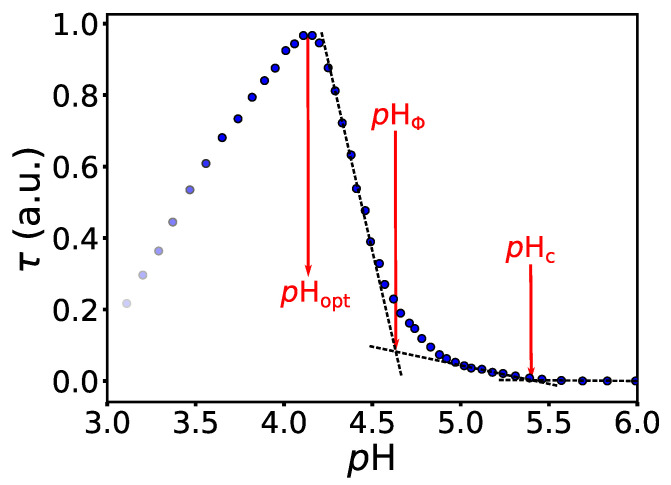
*The turbidimetric titration curve shows the onset of formation of soluble and insoluble protein-PE complexes and their charge neutralization.* Dependence of turbidity, τ, on *p*H for a solution containing 100 μM BSA and 4000 μM NaPSS dissolved in acetate buffer with initial *p*H = 5.8 (I=0.1 M) at 25 ${}^{{}^{\circ}}C$. The determination of the critical *p*H parameters corresponding to the formation of soluble ($pH_{c}$) and insoluble protein-PE complexes ($pH_{\Phi }$) and charge neutralization of the formed complexes ($pH_{\mathrm{opt}}$) is schematically illustrated.

We begin our discussion of the interaction between BSA and NaPSS for the protein-PE system without co-solutes. We use these results as a basis for comparing the effect of added co-solutes. The effects of salts, PEGs, and sugars on the BSA-NaPSS interaction are evaluated mainly by the changes they have on the critical *p*H parameters ($pH_{c}$, $pH_{\Phi }$, $pH_{\mathrm{opt}}$) determined from the turbidimetric curves (cf. Figure 2). A possible explanation for the modulating effect of co-solutes is given, either based on the literature or additional experimental and theoretical techniques (fluorimetry, MD simulations).

### 4.1. The BSA/NaPSS Solutions without Co-Solute

First, we focus on the BSA/NaPSS mixture without the co-solutes present in the solution. The dependence of the turbidity, τ, on the *p*H of the solution (turbidimetric titration curve) is shown for BSA/NaPSS in Figure 2. The initial *p*H of the solution containing 100 μM BSA and 4000 μM NaPSS (r=40) in acetate buffer with 0.1 M ionic strength was 5.8 and was gradually decreased by titration with 0.2 M HCl. The determination of three critical *p*H parameters is also shown schematically in Figure 2. Critical structure-forming events associated with the formation of soluble ($pH_{c}$) and insoluble complexes ($pH_{\Phi }$) were determined as the intersections of two corresponding tangents to the τ vs. *p*H curves [57,58,59]. The *p*H value representing the charge neutralization of the complexes [59,60,61] ($pH_{\mathrm{opt}}$) was determined as the turbidity maximum of the titration curve. By repeating the experiment 3 times, the uncertainty of the critical *p*H parameters was estimated to be ±0.05 (the error was calculated as the largest difference of a single measurement from the mean). It was found that the error for solutions with added salt, PEG, or sugar was not significantly different from the error estimate in (co-solute)-free solution.

Soluble complex formation is initiated at $pH_{c}\approx 5.4$, where both macromolecules (BSA, PSS${}^{-}$) carry a net negative charge. The association of a polyanion with the protein at *p*H values above its isoionic point of BSA (*p*I${}_{\mathrm{BSA}}$≈ 4.7 [56]) is known as the “complexation on the wrong side” [23,26]. The reason for this counterintuitive phenomenon is multifaceted. The charge distribution on the protein surface is not uniform. At short distances, the attractive interaction between the positively charged patches of the protein and the negative polyions may dominate over the repulsive charge-charge interactions, leading to favourable complex formation. On the other hand, entropically driven favourable complex formation is associated with the release of condensed PE counterions upon interaction with the protein. Chodankar et al. [35] performed SANS measurements on BSA/NaPSS mixtures (r≈90 when converting the NaPSS concentration to monomol/L; note also that in Ref. [35] authors define the concentration ratio as r=[BSA]/[NaPSS]) and concluded that soluble complexes occur at *p*H = 7.5, which is well above the *p*I${}_{\mathrm{BSA}}$. Due to the detection limit of the UV-Vis spectrophotometer used in our experiments, as well as different experimental conditions from those in Ref. [35] (presence of NaCl, MW of NaPSS was 100 kDa), our estimates for $pH_{c}$ are lower. However, we were not so much interested in the absolute values of the critical parameters, but in the relative changes of these parameters with the addition of co-solutes.

The formation of insoluble BSA/NaPSS complexes was estimated to occur at $pH_{\Phi }$≈ 4.6. Of course, the turbidity of the solution begins to increase before this *p*H value, which makes it difficult to accurately determine $pH_{\Phi }$. However, throughout the work, all $pH_{\Phi }$ values were determined in the same way (as shown in Figure 2), and again, it is the relative changes with respect to the addition of co-solute that are of most interest and will be discussed in the subsequent sections.

After further acidification of the solution, the turbidity reaches a maximum value at $pH_{\mathrm{opt}}\approx 4.2$. At this point the net charge of the complexes is zero [59,60,61]. The decrease in turbidity below $pH_{\mathrm{opt}}$ is associated with the dissolution of insoluble complexes probably due to the interparticle repulsion between like-charged particles.

From a macroscopical standpoint the complex formation between BSA and NaPSS that occurs when the *p*H value is lowered leads to the formation of precipitates (solid-liquid phase separation). Figure S1 shows a photograph of the solution taken with an optical microscope. Small solid-like particles can be seen at the point of maximum turbidity ($pH_{\mathrm{opt}}$≈ 4.2) for different NaPSS to BSA molar ratios (r=40,45, and 50). However, the amount of solid particles at the chosen molar ratio (r=40) is very low and the light scattering is attributed mostly to the highly hydrated particles due to a large number of free (uncomplexed) charges on the protein surface. The solid-liquid nature of phase separation is clearly visible at higher molar ratios (r=50). Since the presence of different co-solutes can either promote or hinder the formation of protein-PE complexes, the molar ratio of r=40 was chosen as a reference in all further experiments.

Considering that the charge of NaPSS is *p*H-independent and that the interaction between BSA and NaPSS is caused by the changing charge distribution on the protein surface, the modulating effect of co-solutes should be a consequence of protein-(co-solute) interactions.

### 4.2. Salt-Specific Influence on BSA/NaPSS Complexation

**Figure 3:**
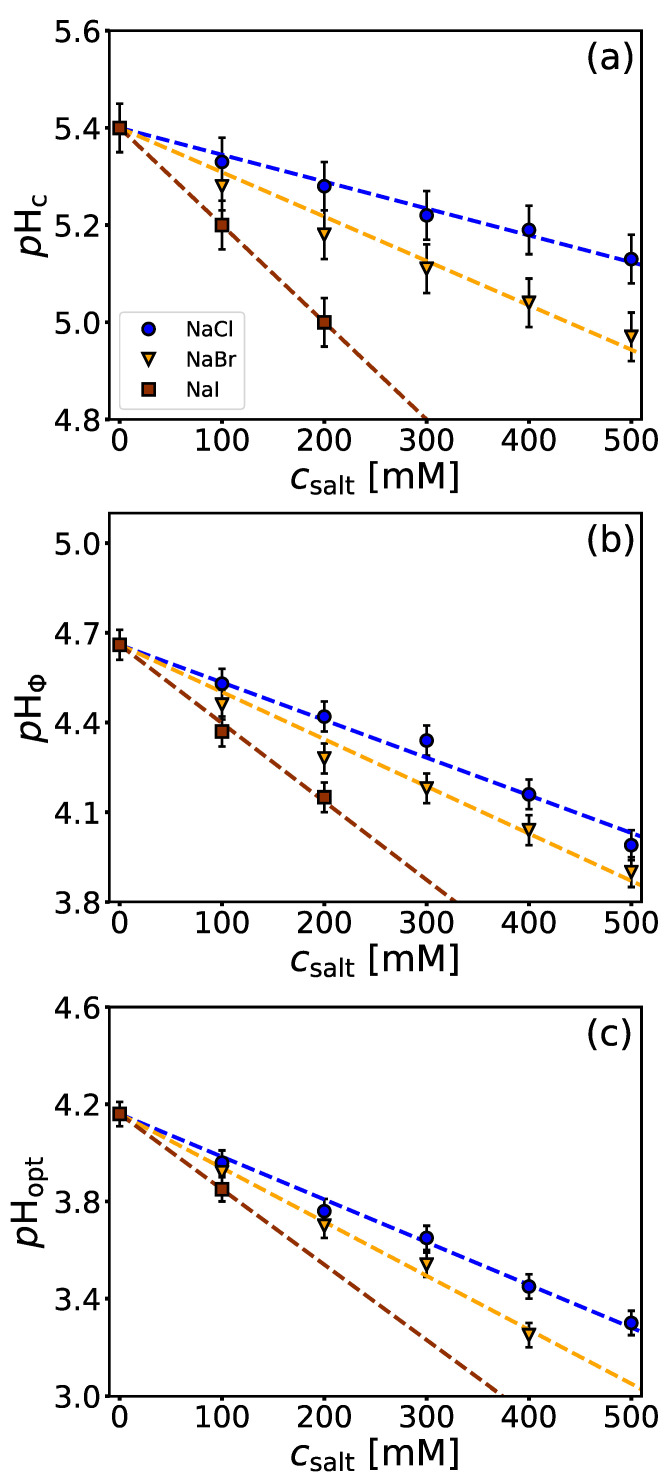
*The presence of salt hinders critical structure forming events due to electrostatic screening.* Critical *p*H parameters (**a**) $pH_{c}$, (**b**) $pH_{\Phi }$, and (**c**) $pH_{\mathrm{opt}}$ as a function of salt concentration. Parameters were extracted from turbidimetric titration curves obtained by the acidification of BSA/NaPSS solutions (r=40) in acetate buffer (*p*H = 5.8, I=0.1 M) containing NaCl, NaBr, and NaI at 25 ${}^{{}^{\circ}}C$ (cf. Figure S2).

In general, addition of salts greatly affects the complex formation between the protein and the PE, mainly as a consequence of electrostatic screening. The effect depends both on the salt concentration as well as on the salt type [38]. We investigated the influence of NaCl, NaBr, and NaI on the critical *p*H parameters. The dependedencies of these parameters, extracted from turbidimetric curves (Figure S2, left), are shown in Figure 3 as a function of salt concentration.

Figure 3a shows that the presence of salt in the solution decreases the value of $pH_{c}$. This means that salt hinders the formation of soluble complexes, which is not surprising since $pH_{c}$ usually depends only on the ionic strength of the solution [1]. The formation of insoluble complexes ($pH_{\Phi }$) is also deterred by the presence of salts (Figure 3b). The stabilizing character of salts as a function of increasing concentration is a consequence of the screening of attractive electrostatic interactions between BSA and NaPSS. This is mainly associated with a decrease in entropy gain as the release of counterions is hindered in the presence of salts [22,62,63]. Within the scope of the paper we focus on higher ionic strengths (I>100 mM), however one should keep in mind that the presence of salt ions at lower ionic strengths (I≲20 mM) may actually promote phase separation [29]. The *p*H of charge neutralization of the complexes ($pH_{\mathrm{opt}}$) also decreases with increasing salt concentration (Figure 3c), again pointing at screening of the attractive forces between BSA and NaPSS molecules. Therefore, a higher net positive charge of BSA (lower medium *p*H) is needed to achieve charge neutrality of the complexes.

**Figure 4:**
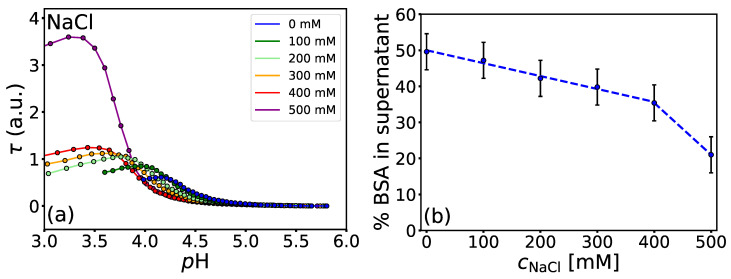
*The presence of salts affects protein-PE complexation as well as protein aggregation.* (**a**) Turbidimetric curves for the titration of 100 μM BSA and 4000 μM NaPSS in the presence of different NaCl concentrations and (**b**) the concentration of BSA in the supernatant measured after centrifugation of the solutions titrated only to $pH_{\mathrm{opt}}$ determined at 25 ${}^{{}^{\circ}}C$.

From the turbidimetric curves (Figure S2), one can see that even at low *p*H values (*p*H ≲*p*I${}_{\mathrm{BSA}}$), turbidity increases with increasing salt concentration for a given *p*H. This suggests that the presence of salts enhances phase separation. It has been shown that the cloud point temperatures of lysozyme [64] and BSA [65] solutions increase with increasing concentration of NaCl at pH<pI, implying that formulations with higher salt content are less colloidally stable. Figure 4a shows the turbidimetric curves for BSA/NaPSS (r=40) solutions with different amounts of NaCl. An abrupt change in τ at $pH_{\mathrm{opt}}$ is observed when the concentration of NaCl is increased from 400 to 500 mM. To estimate the fraction of uncomplexed BSA at $pH_{\mathrm{opt}}$ for a given NaCl concentration (up to 500 mM), the turbidimetric titrations were repeated, stopping the acidification when $pH_{\mathrm{opt}}$ was reached. Each solution was then transferred to a microcentrifuge tube, centrifuged at 10,000 RPM for 20 min, and the absorbance of the supernatant measured at 280 nm. From this information, the percentage of BSA in the supernatant was determined as a function of NaCl concentration (Figure 4b). Since the amount of uncomplexed NaPSS in the supernatant is not precisely known, the uncertainty of the determined BSA concentration was estimated from the absorbance of a solution containing 4000 μM NaPSS alone. As can be seen from Figure 4b, less BSA remains in the supernatant with increasing salt concentration, which also correlates with the trends in τ. The increase in turbidity at low *p*H values is probably a consequence of BSA aggregation, approaching the phase stability limit of the solution at the temperature of the experiment when the NaCl concentration is increased above 400 mM. The SANS study by Chodankar et al. [35] also showed that increasing the ionic strength of the solution affected not only the interaction between BSA and NaPSS but also between protein molecules. They found that the size of the complexes remained approximately the same with increasing ionic strength, but the distances between cross-linked points in the primary complex tended to decrease. The latter refers to proteins that act as crosslinkers for PE chains. In addition, the increase in turbidity (Figure 4a) could also be explained by aggregation of the primary protein-PE complexes at higher salt concentrations, as explained by Dautzenberg et al. [37], although most likely several simultaneous effects occur as a result of modulation by the co-solute.

In addition to the concentration-dependent stabilizing effect of salts, the chemical identity of the salt anion also plays an important role. As shown in Figure 3, the stabilizing effect of a salt increases in the order: NaCl < NaBr < NaI, which is consistent with the so-called inverse Hoffmeister series for anions. Salt-specific effects have been previously observed in regard to the formation of polyelectrolyte multilayers between NaPSS and various polycations (PAH [66], PDADMAC [67], and PDADMA [68]). Thicker multilayers are expected in the presence of larger and more polarizable anions (I${}^{-}$ and Br${}^{-}$) that adhere to polycations. This shields the repulsive interactions between positively charged groups of the polycation, allowing PEs to adopt a more coiled conformation that manifests itself in a thicker multilayer build-up. Similarly, the heterogeneous charge distribution on the protein surface leads to the formation of negative and positive patches which interact with charged species.

Ion-specific effects observed in critical *p*H parameters can be interpreted in light of Collin’s law of matching water affinities [69,70]. Charged patches on the protein surface consist mostly of protonated amine groups ($-{\mathrm{NH}}_{3}^{+}$) and deprotonated carboxylic groups ($-{\mathrm{COO}}^{-}$), where the former can be classified as chaotropic and the latter as cosmotropic [71]. The affinity of salt anions for the $-{\mathrm{NH}}_{3}^{+}$ groups follows the order: I${}^{-}$> Br${}^{-}$> Cl${}^{-}$ (from more to less chaotropic) [72]. Such a tendency of anions for BSA was seen from the mixing enthalpies determined by an ITC calorimetry study of BSA-salt mixtures [64]. The heat effects were correlated with the enthalpies of hydration of the salt anions, indicating a higher affinity of the chaotropic anions for BSA. Recently, Džudžević-Čančar et al. [65] showed that phase separation in BSA-PEG-salt mixtures depends on the chemical identity of the salt anions (and cations) as well as on the *p*H of the medium. Consistent with the law of matching water affinities, more chaotropic salt anions are more effective in “neutralizing” positive charges ($-{\mathrm{NH}}_{3}^{+}$) on the protein surface. This effectively increases the net negative charge on the protein surface at $pH>pI_{\mathrm{BSA}}$ and decreases the net positive charge on the protein at $pH<pI_{\mathrm{BSA}}$. This results in greater repulsion between BSA molecules and between BSA and NaPSS molecules above the isoionic point, but leads to a stronger attraction between BSA molecules and a weaker attraction between BSA and NaPSS moleules below the isoionic point. Looking at the turbidity curves (Figure S2, left), we can see that the absolute value of turbidity (change with respect to the *y*-axis) increases with salt concentration, and more noticeably in the presence of more chaotropic salt anions (I${}^{-}$, Br${}^{-}$) and below the isoionic point. Due to the complex nature of BSA-NaPSS salt solutions, BSA aggregation and BSA/NaPSS complexation occur simultaneously with a change in *p*H. However, the aggregation of protein molecules is evident when the titration is performed without NaPSS (cf. Figure S4 showing turbidimetric curves for BSA-NaI solutions).

### 4.3. Influence of Molecular Crowders on BSA/NaPSS Complexation

The presence of molecular crowders (e.g., PEG) in protein solutions often reduces the effective volume available to the proteins which in turn affects protein-protein interactions. The molecular mechanism by which PEG has an effect on (bio)macromolecules is a consequence of the exclusion of PEG molecules from the region between colloids, resulting in a depletion attraction [73]. PEG promotes the agregation of BSA in solutions with or without salt and the effect is stronger in case of PEGs having larger molecular masses [65]. In addition, the hydrophilicity of PEG brings about the dehydration of proteins [74]. When it comes to the effect of PEG on the complex coacervation of oppositely charged polyelectrolytes, PEG promotes the complex formation [75,76]. Although PEG is perceived as an inert molecule, weak protein-PEG interactions have been documented in the literature [77,78]. We used fluorescence quenching analysis to elucidate possible contributions of PEG interactions with the hydrophobic parts of the BSA surface.

**Figure 5:**
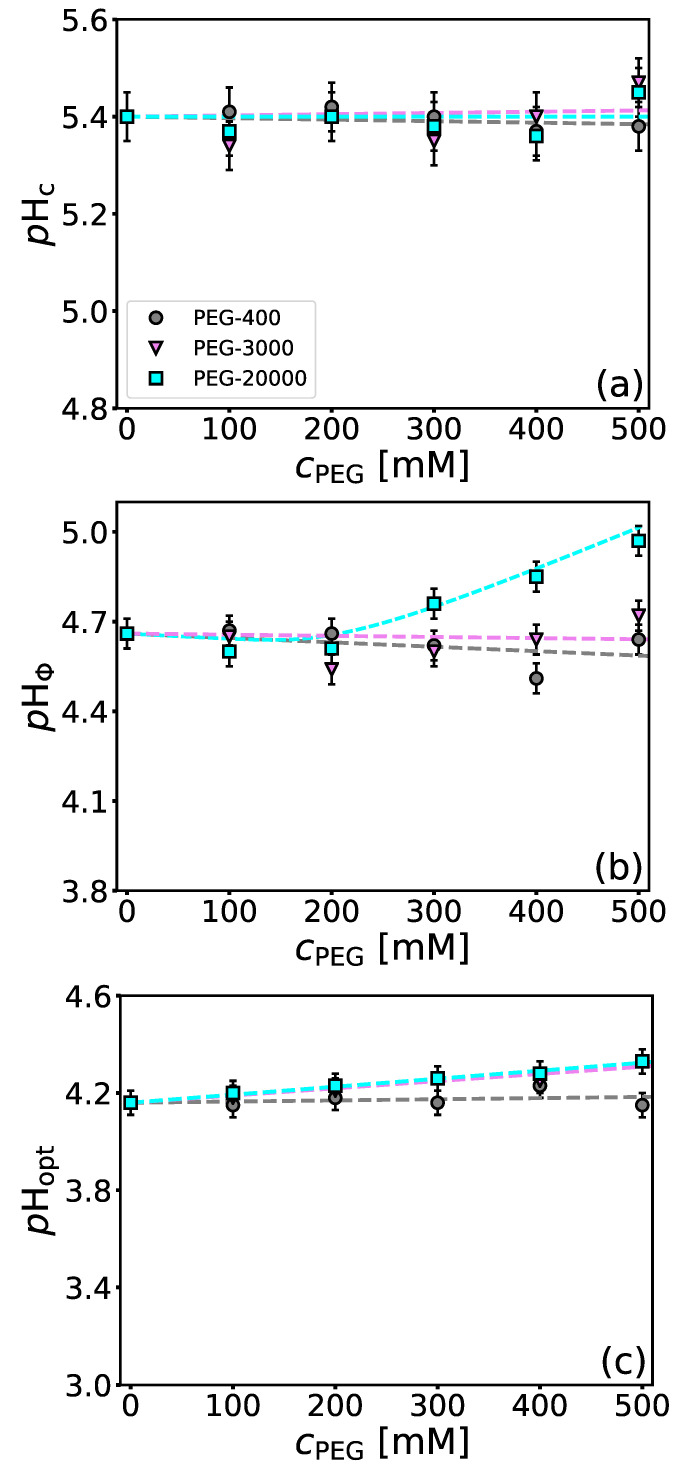
*Only high-MW PEG at high concentrations promotes the formation of insoluble protein-PE complexes.* Same as in Figure 3 but for PEG-400, PEG-3000, and PEG-20000: (**a**) $pH_{c}$, (**b**) $pH_{\Phi }$, and (**c**) $pH_{\mathrm{opt}}$.

We have tested the influence of PEG-400, PEG-3000, and PEG-20000 on the critical *p*H parameters. As can be seen from Figure 5a, the presence of PEG of all evaluated molecular weights until 500 mM does not have any noticeable effect on the formation of soluble complexes (no significant changes in $pH_{c}$ as a function of PEG concentration and MW was observed). For PEG-400 and PEG-3000 (as well as for PEG-20000 up to 200 mM), a similar observation can be made also in case of insoluble complexes ($pH_{\Phi }$, Figure 5b). However, in the case of PEG-20000 an increase in $pH_{\Phi }$ upon PEG concentration above 200 mM can be seen. This implies that PEG-20000 at higher concentrations promotes insoluble complex formation (Figure 5b). High-MW PEG has often been documented to bind to hydrophobic pockets of proteins [41,42]. We measured the fluorescence spectra of BSA in the presence of different PEG concentrations for higher-MW PEGs (PEG-3000, PEG-20000) and performed a fluorescence quenching analysis (Stern-Volmer plots) to elucidate possible protein-PEG interactions (the emission spectra are given in Figure S6 in the Supplementary Materials file). The values of Stern-Volmer constants, $K_{\mathrm{SV}}$ (cf. Equation (2)), are for BSA solutions with PEG-3000 and PEG-20000 given in Table 1 for *p*H = 4.2 and *p*H = 5.8. The quenching is minimal, with the values of $K_{\mathrm{SV}}$ with respect to the *p*H or MW of PEG being within the experimental uncertainty. Considering that the interactions between BSA and NaPSS are predominantly electrostatic, weak BSA-PEG interactions can be neglected in considering BSA/NaPSS complexation.

The effect of PEG on the *p*H parameter related to the charge neutralization of complexes ($pH_{\mathrm{opt}}$) is shown in Figure 5c. In case of PEG-400 no effect of the polymer on the $pH_{\mathrm{opt}}$ was observed in the investigated concentration range. For PEGs with higher-MW a slight shift to larger *p*H values was observed. This could be a consequence of depletion interactions, which induce the neutralization of the complexes at higher *p*H values (Figure 5c).

As can be seen from turbidimetric curves in Figure S2 (right) the presence of PEG-400 causes a slight increase in the absolute turbidities in a concentration dependent manner. The rapid rise of turbidities in the presence of PEG-3000 and PEG-20000 is probably analogous as with the presence of NaBr and NaI in that the presence of higher-MW PEG causes more protein molecules to aggregate. The rise in τ is a consequence of complexation and aggregation, meaning that more protein molecules are precipitated from the solution in the presence of PEG.

### 4.4. Influence of Sugars on BSA/NaPSS Complexation

**Figure 6:**
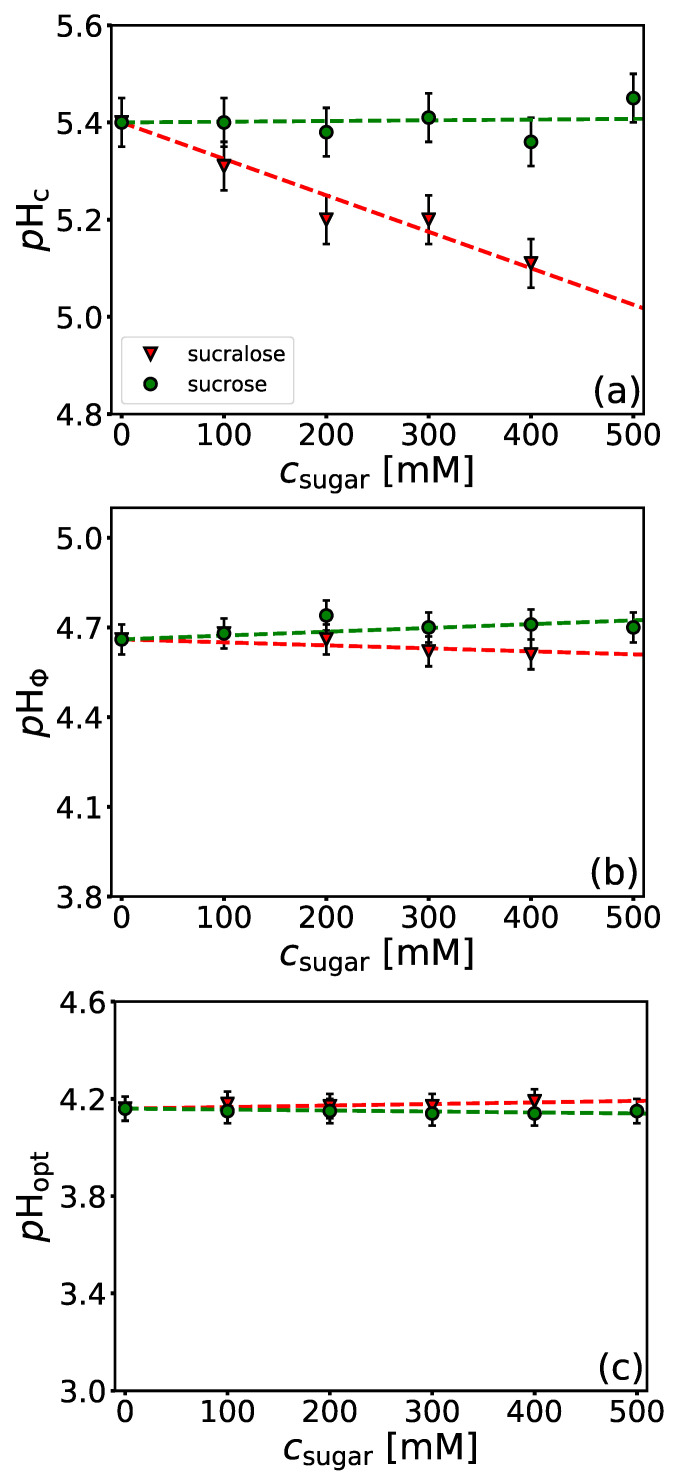
*Sucralose hinders the formation of soluble protein-PE complexes.* Same as in Figure 3, but for sucrose and sucralose: (**a**) $pH_{c}$, (**b**) $pH_{\Phi }$, and (**c**) $pH_{\mathrm{opt}}$.

Sugars such as sucrose, trehalose, and sorbitol are known for their bioprotective abilities. Due to their stabilizing nature, sugars are often included in pharmaceutical formulations. In this work, we investigated the effect of two structural analogues, sucrose and sucralose (see Figure 1c,d), on BSA-NaPSS complexation. Sucralose (a commonly used artificial sweetener in foods) is the chlorinated analogue of sucrose (3 OH groups are substituted by 3 Cl atoms). The chemical modification of sucrose leads to different physico-chemical properties and water structuring capabilities of these two sugars [43]. By studying the effects of sucrose and sucralose on the phase stability of lysozyme solutions, we have shown that sucralose has a greater propensity to the protein surface, in contrast to sucrose, which is preferentially excluded from it [53]. Thus, sucralose acts as a better stabilizing agent by preventing protein molecules from coming into contact (aggregating). Figure 6a shows the dependence of $pH_{c}$ on sugar concentration. We see that the soluble complex formation is not affected by the presence of sucrose, while the formation is hindered in the presence of sucralose. Similar observations apply also to the formation of the insoluble complexes (Figure 6b). There is no influence of sucrose on $pH_{\Phi }$ in the whole concentration range studied, while sucralose slightly hinders the formation of complexes. The influence of sucralose is less pronounced here than in modulating the formation of the soluble BSA/NaPSS complexes. As can be seen from Figure 6c, the charge neutralization of the complexes is not affected by the presence of sugars. Since BSA/NaPSS complex formation is electrostatic in nature, uncharged sugar molecules are not expected to affect the $pH_{\mathrm{opt}}$. The turbidimetric curves are shown in Figure S3. The absolute values of the turbidities at $pH_{\mathrm{opt}}$ decrease slightly in the presence of sucrose and sucralose. This is probably the result of the bioprotective nature of sugars, which prevent protein-protein aggregation.

**Figure 7:**
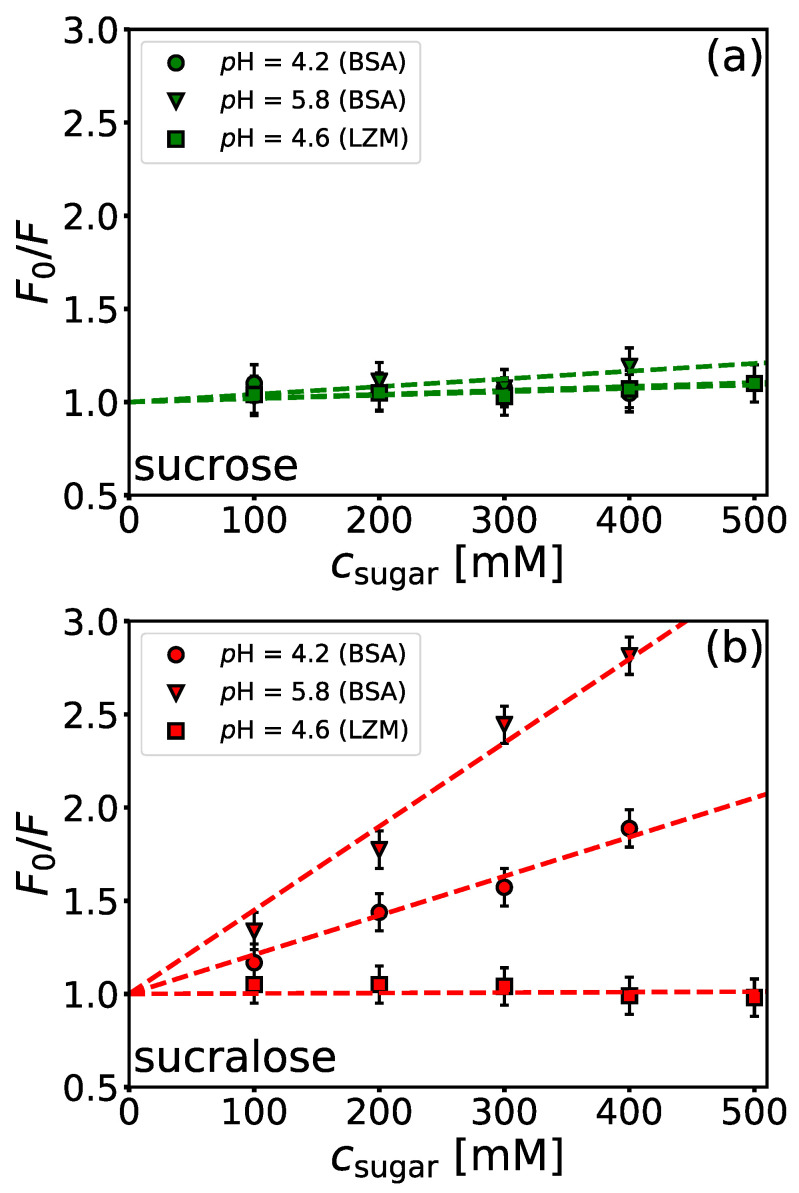
*Sucralose interacts with hydrophobic pockets of BSA.* Stern-Volmer plots for the quenching of BSA and lysozyme (LZM) by (**a**) sucrose and (**b**) sucralose. The concentration of BSA and LZM was 0.5 and 2 μM, respectively ($\lambda_{\mathrm{ex}}=280$ nm, T=25 ${}^{{}^{\circ}}C$). All solutions were prepared in acetate buffers of different *p*H-values but with the same ionic strength (I=0.1 M).

Presented data speak in favour of the stabilizing role of sucralose in BSA/NaPSS solutions, especially at the onset of the formation of the soluble protein-PE complexes. At $pH>pI_{\mathrm{BSA}}$, the interactions between BSA and NaPSS are rather weak due to electrostatic repulsion, but soluble complexes can still be formed due to the interaction between negatively charged PE and positively charged patches on the protein surface (“complexation on the wrong side”, see beginning of Results and Discussion). In addition to the charged sulfonic functional groups responsible for the charge-charge interactions, the repeating unit of polystyrene sulfonate contains a hydrophobic aromatic phenyl ring (Figure 1a). These parts of the NaPSS molecule can lead to non-negligible hydrophobic contacts with the lipophilic pockets of BSA. The interaction of sucralose with the hydrophobic pockets of BSA was recently studied by fluorescence quenching [79], but at lower sugar concentrations than used in this work. We performed a quenching assay to evaluate the interaction of sucrose and sucralose with BSA at two different *p*H values (above and below the isoionic point), where BSA has a different net charge (*p*H = 4.2 and *p*H = 5.8). The emission spectra are given in Figure S8 in the Supplementary Materials file. CD measurements (Table S1) indicated that sugars do not affect the structural state of BSA, indicating that the quenching is a consequence of direct BSA-sugar interactions (see comment in Section S5 of the Supplementary Materials file). A Stern-Volmer plot is given in Figure 7 and the corresponding quenching constants, $K_{\mathrm{SV}}$, are collected in Table 2. For comparison, results for hen-egg white lysozyme are also given. As can be seen from Figure 7a, the interaction of sucrose with hydrophobic parts of the BSA surface is negligible for both *p*H values. Although there are marked differences in the quenching constants (Table 2) at both *p*H values, the values of $K_{\mathrm{SV}}$ are too small to denote an interaction of sucrose with the hydrophobic parts of the protein surface. In contrast to the non-interacting nature of sucrose, sucralose shows a greater decrease in fluorescence intensity (Figure 7b). This indicates that it adheres to the hydrophobic parts of BSA. The effect is more pronounced at pH=5.8, where BSA is net negatively charged, than at pH=4.2, where BSA is net positively charged. The value of $K_{\mathrm{SV}}$ at pH=5.8 is more than twice that at 4.2, which could be a consequence of sucralose interacting not only with hydrophobic pockets of BSA but also with negatively charged amino acid residues on the protein surface (see Ref. [53]). Note that at pH=5.8, the number of COO^−^ residues on the protein surface is greater than at 4.2. We also performed a similar analysis for lysozyme solutions (*p*H = 4.6, I=0.1 M)—for emission spectra see Figure S8. At this pH, lysozyme is net positively charged. As with BSA, the data suggest that sucrose does not interact with the hydrophobic parts of the lysozyme surface (cf. Figure 7a). However, in contrast to BSA, sucralose also showed no quenching effect (cf. Figure 7b). Lysozyme is a much smaller protein compared to BSA (MW of lysozyme is 14.3 kDa), and the molecule is more spherical. It has a more uniform distribution of ionizable amino acid residues on its surface and is known not to have significant amounts of hydrophobic patches. Our results suggest that the mechanism of sucralose-BSA interaction should also be considered protein-specific. In the case of BSA, the effect on the formation of the BSA/NaPSS complex depends on the accessibility of the hydrophobic residues to sucralose molecules.

To supplement experimental findings, sugar-protein solutions in explicit water (SPC/E) have been explored computationally. We performed MD simulations of BSA in the presence of sucrose/sucralose for two *p*H values (*p*H = 4.2 and *p*H = 5.8). The propensity of sugars toward the protein surface was quantified by calculating the average number of sugar molecules around the protein, 〈N(r)〉. The minimum distance between the protein surface atom (including hydrogens) and the closest oxygen atom of each sugar molecule was considered. For each time frame the distribution was calculated up to 3 nm away from the protein surface, and collected in 0.01 nm wide bins. The resulting distribution of sugar molecules around the protein was then averaged over all time frames.

**Figure 8:**
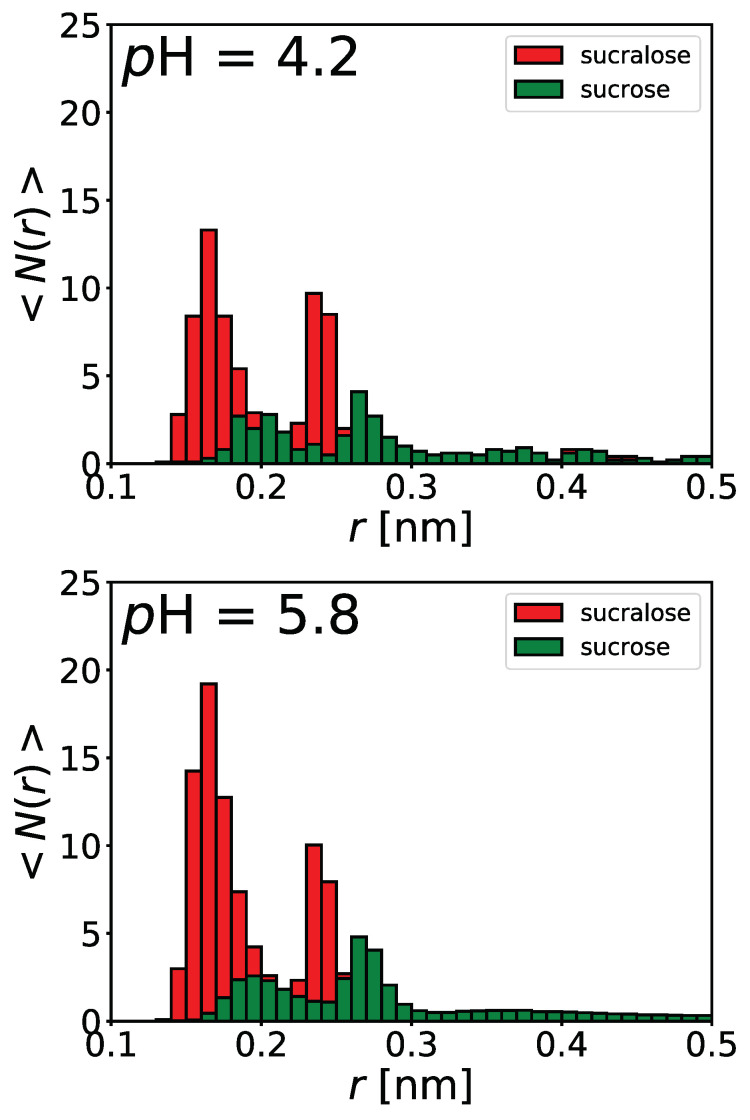
*Sucralose has a greater propensity towards the protein surface.* Average number of closest sugar atoms from the closest protein atom (hydrogens included) in the vicinity of the protein surface grouped into 0.01 nm wide bins. Only the closest oxygen atom of each sugar molecule was considered. All for 25 ${}^{{}^{\circ}}C$.

In Figure 8 the 〈N(r)〉 is shown. We see that at both pH values, the interaction of sucralose with the protein is much stronger than that of sucrose. The two peaks observed in the 〈N(r)〉 for both sugars correspond to the hydrogen bonds (HBs) formed between the sugar molecules and the protein surface. The first peak is centered at about 0.165 nm for sucralose and 0.195 nm for sucrose. This peak corresponds to hydrogen bonds where the surface residues act as HB donors. The second peak centered at about 0.235 nm for sucralose and 0.265 nm for sucrose corresponds to HBs formed when the surface residues act as HB acceptors. Comparing the propensity of sucralose to BSA at both pH values, we can see that more sucralose molecules gather around the protein surface at pH=5.8, while no noticeable difference is seen for sucrose. Since changes in the *p*H value of the medium affect the protonation states of the acidic/basic amino acid residues at the protein surface, this difference could be explained by the fact that sucralose molecules are in close proximity to both hydrophobic as well as charged residues, the latter having $pK_{a}$ values around the isoionic point of BSA (glutamic acid (GLU) has a $pK_{a}\approx$ (4.2–4.4)). By plotting the 〈N(r)〉 only for glutamic acid (and for hydrophobic tryptophan (TRP)) we were able to elucidate that more sucralose molecules accumulate around GLU residues at pH=5.8 than at pH=4.2 (Figure S9). At pH=5.8, the amino acid residues are deporotonated and the carboxylate groups (COO${}^{-}$) act as stronger HB acceptors, while at 4.2 only a portion of these groups are in ionic form. MD simulations, however, do not show any significant clusters of sugar molecules around the TRP residues. This is likely a consequence of the sampling (BSA has only two TRP residues as opposed to 59 GLU residues), as well as the fact that less favourable van der Waals forces may be underrepresented by the force field used. Nevertheless, the results of the fluorimetry measurements and the MD simulations should be considered complementary and not contradictory.

To better highlight the local distribution of water (and indirectly sugar) molecules around the protein surface, we calculated the time-averaged normalized ratio ($G_{\mathrm{ow}}$) as a function of distance from the protein surface, *r* (see Supplementary Material and Figure S10 for details and results). If $G_{\mathrm{ow}}>1$, this indicates that the protein surface is preferentially hydrated. However, if $G_{\mathrm{ow}}<1$, the sugar is preferentially bound to the protein surface. The $G_{\mathrm{ow}}\left(r\right)$ given in Figure S10 shows that sucralose interacts strongly with the BSA surface, whereas sucrose does so to a minor extent. The same conclusion was drawn from our recent work on the interactions between lysozyme and sugars [53]. Taking into account experimental measurements (Figure 6 and Figure 7) as well as MD simulations, we can conclude that sucralose adheres to negatively charged amino acid residues on the BSA surface as well as to its hydrophobic pockets. Since the latter is probably partly responsible for modulating the interaction between BSA and NaPSS at $pH>pI_{\mathrm{BSA}}$, we assume that sucralose acts as a competitor of NaPSS for BSA.

In general, sugars do not have a major impact on the stability of protein-PE complexes in aqueous solutions, but as shown in this work, their chemical modification can be considered as a way to achieve better modulation even in systems where electrostatic interactions are the predominant driving force of complexation.

## 5. Conclusions

The effect of three types of co-solutes (salts, PEGs, and sugars) on the complexation between bovine serum albumin and sodium polystyrene sulfonate was studied by turbidimetric titrations. Critical structure-forming parameters associated with the formation of soluble ($pH_{c}$) and insoluble complexes ($pH_{\Phi }$) as well as the charge neutralization of the complexes ($pH_{\mathrm{opt}}$) were determined at different co-solute concentrations. From a macroscopic point of view, the interaction between BSA and NaPSS leads to the formation of solid particles under the conditions studied. The presence of evaluated co-solutes does not seem to affect the nature of phase separation.

The presence of salts hinders the formation of soluble and insoluble complexes as well as the charge neutralization of the complexes. All effects can be attributed to the screening of electrostatic interactions between BSA and NaPSS. The chemical identity of the salt anion also plays an important role. The stabilizing effect of a salt increases in the order: NaCl < NaBr < NaI. In addition, the presence of salts also affects protein-protein interactions, resulting in BSA/NaPSS complexation being accompanied by protein aggregation. This is more evident in the presence of more chaotropic salt anions (I^−^, Br^−^) and below the isoionic point of the protein.

Polyethylene glycol has no effect on the formation of soluble complexes regardless of its molecular weight. However, PEG with a high molecular weight (20,000 g/mol) and at sufficiently high concentrations (above 200 mM) showed a tendency to promote the formation of insoluble complexes due to depletion interactions. The effect of PEG on the charge neutralization of the complex was observed for PEG-3000 and PEG-20000 and is probably caused by the same mechanism.

In contrast to the stabilizing nature of sugars with regard to the phase stability of protein-sugar solutions, the presence of sucrose does not affect the formation of soluble/insoluble complexes or their charge neutralization. Sucralose, on the other hand, inhibits the formation of soluble complexes as well as slightly deters the formation of insoluble complexes. The reason for this difference is probably a greater propensity of sucralose towards the hydrophobic pockets of BSA. We assume that sucralose acts as a competitor to NaPSS when it comes to the formation of BSA/NaPSS complexes.

Our results shed light on the complex interactions in multicomponent solutions by evaluating the effects of different co-solutes on the interactions between simple model systems (BSA, NaPSS) with well-established physico-chemical properties. These findings are beneficial for the preparation of multicomponent protein formulations, which are the focus of protein drug formulations, as well as for other biotechnological applications such as protein purification by precipitation.

## Figures and Tables

**Table 1:** Stern-Volmer quenching constants ($K_{\mathrm{SV}}$) in L mol${}^{-1}$ for BSA in the presence of PEG-3000 and PEG-20000 at *p*H = 4.2 and *p*H = 5.8 (T=25 ${}^{{}^{\circ}}C$).

Co-Solute	*p*H = 4.2	*p*H = 5.8
PEG-3000	0.15 ± 0.08	0.15 ± 0.08
PEG-20000	0.22 ± 0.06	0.17 ± 0.03

**Table 2:** Stern-Volmer quenching constants ($K_{\mathrm{SV}}$) in L mol${}^{-1}$ for BSA (*p*H = 4.2 and *p*H = 5.8) and lysozyme (LZM; *p*H = 4.6) in the presence of sucrose and sucralose.

	BSA	BSA	LZM
Co-Solute	pH = 4.2	pH = 5.8	pH = 4.6
sucrose	0.21 ± 0.09	0.41 ± 0.07	0.18 ± 0.02
sucralose	2.10 ± 0.09	4.5 ± 0.2	0.02 ± 0.06

## Data Availability

The data presented in this study are available upon request from the corresponding author.

## Supplementary Materials

The following supporting information can be downloaded at: https://www.mdpi.com/article/10.3390/polym14061245/s1, Figure S1: turbidimetric titration curves at different molar ratios of NaPSS to BSA (r=40, 45, and 50) and photographs of solutions at $pH_{\mathrm{opt}}$ taken with an optical microscope, Figures S2 and S3: turbidimetric titration curves of all BSA–NaPSS–(co-solute) solutions, Figures S4 and S5: turbidimetric titration curves of BSA-NaI and BSA-NaPSS-NaI solutions and microscopy images for these two cases at $pH_{\mathrm{opt}}$, Figures S6 and S7: emission spectra of BSA-PEG solutions (PEG-3000, PEG-20000) and subsequent Stern-Volmer plots at *p*H = 4.2 and *p*H = 5.8, Table S1: mean residue ellipticities of BSA-sugar solutions at 222 nm, Figure S8: emission spectra of BSA-sugar solutions at *p*H = 4.2 and 5.8 as well as LZM-sugar solutions at *p*H = 4.6, Figure S9: average number of sugar oxygen atoms around glutamic acid and tryptophan residues, Figure S10: the local distribution of sugar/water molecules around the protein surface, $G_{\mathrm{ow}}\left(r\right)$. Section S1. Phase separation in the BSA/NaPSS system is solid-liquid; Section S2. Turbidimetric curves for BSA/NaPSS solutions with added salt, PEG or sugar; Section S3. BSA/NaPSS complexation is accompanied by BSA aggregation; Section S4. Interaction of PEG with hydrophobic surface of BSA is negligible; Section S5. Interaction of sugars with hydrophobic surface of BSA; Section S6. Local distribution of sugar and water molecules around the protein surface.

## Abbreviations

The following abbreviations are used in this manuscript:

PE	polyelectrolyte
BSA	bovine serum albumin
NaPSS	sodium polystyrene sulfonate
PEG	polyethylene glycol
