# Supplementary material for: Modulating Role of Co-Solutes in Complexation between Bovine Serum Albumin and Sodium Polystyrene Sulfonate
Source: Polymers (Basel). 2022 Mar 19;14(6):1245. doi: 10.3390/polym14061245 (PMC8953846; doi:10.3390/polym14061245)
Supplement: Supplementary file 1 [file polymers-14-01245-s001.zip › polymers-1629148-supplementary.pdf]

Supplementary Materials: Modulating Role of Co-solutes inComplexation between Bovine Serum Albumin and SodiumPolystyrene Sulfonate

Matjaž Simončič and Miha Lukšič

Section S1. Phase separation in the BSA/NaPSS system is solid-liquid

Figure S1 (top) shows the turbidimetric titration curves for BSA/NaPSS solutions with different NaPSS to BSA concentration ratios r = 40, 45, and 50 (in all cases the concentration of BSA was 100 μM; $r = [\text{NaPSS}]_{\text{monomol}}/[\text{BSA}]$). Initial solution was prepared in acetate buffer with pH = 5.8 and 0.1 M buffer ionic strength, and titrated with 0.2 M HCl. Increasing the concentration of NaPSS (larger r values) promotes the extent of complex formation. The turbidity, τ, at a given pH increases with increasing r, since larger content of NaPSS in solution promotes complexation as well as potential aggregation of protein molecules (see also page 4 of the SI).

Photographic images of the systems at pHopt taken with an optical microscope are shown in Figure S1 (bottom). Acidification of BSA/NaPSS originally dissolved in acetatebuffer (pH = 5.8, I = 0.1 M) leads to the formation of solid particles, but precipitates are difficult to see at r = 40 and 45 (red circles). A lower extent of precipitation at lower r can be explained by the formation of highly hydrated precipitates as a consequence of a larger number of free (hydrated) protein charges due to the molar ratio. When the latter is increased to r = 50, we can observe the formation of "true" precipitates, leading to the conclusion that, from a macroscopic point of view, the interaction between BSA/NaPSS leads to the formation of precipitates rather than coacervates. However, this does not exclude the possibility of formation of coacervates at other physical parameters (higher concentrations of BSA and NaPSS, pH ≈ 5.0).

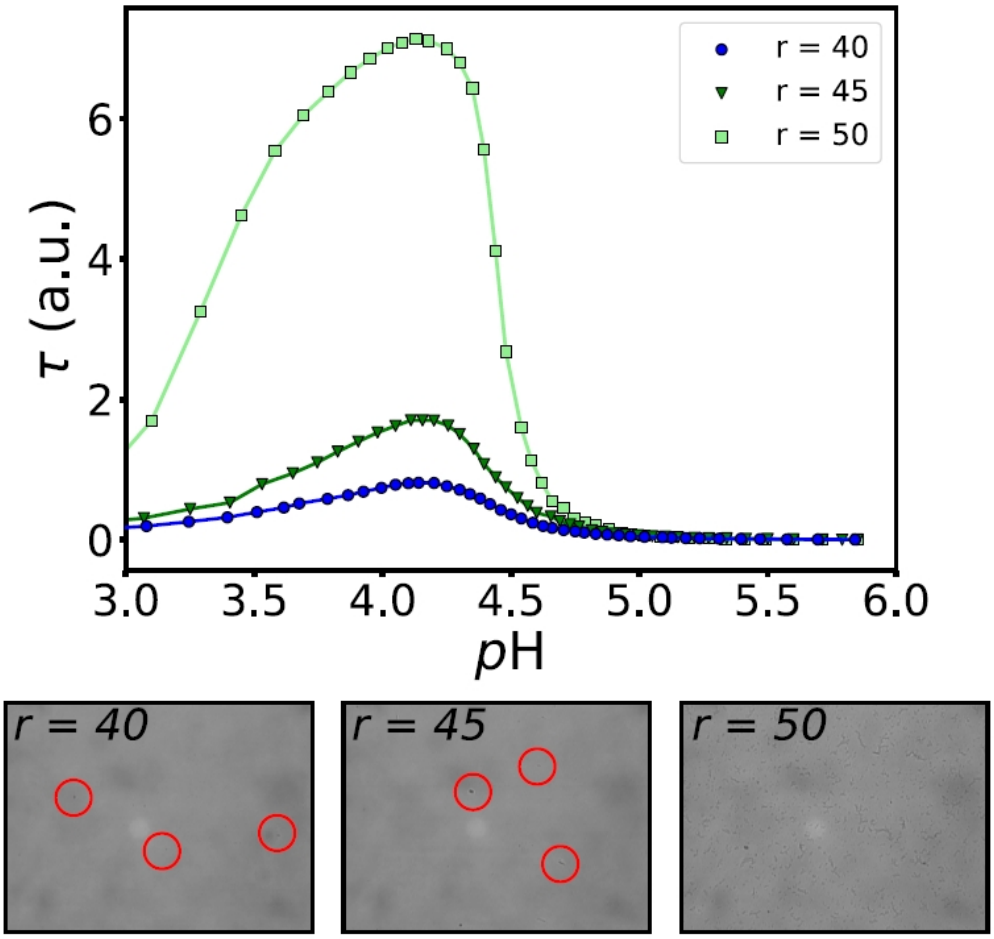

Figure S1. Top: Turbidimetric titration curves of 100 μM BSA and NaPSS (r = 40, 45, 50) dissolved in acetate buffer (pH = 5.8, 0.1 M ionic strength). Bottom: Optical microscopy images at $p\mathrm{H}_{\mathrm{opt}}$ for all three molar ratios. Solid precipitates at r = 40 and 45 are marked with red circles and correspond to highly hydrated protein-PE complexes (T = 25 °C).

Section S2. Turbidimetric curves for BSA/NaPSS solutions with added salt, PEG or sugar

Figure S2 shows turbidimetric titration curves for BSA/NaPSS (r = 40) solutions with added NaCl, NaBr, and NaI (left) or PEG-400, PEG-3000, and PEG-20000 (right) at various concentrations (up to 500 mM). Similarly, Figure S3 shows turbidimetric titration curves for BSA/NaPSS solutions with added sucrose (left) and sucralose (right).

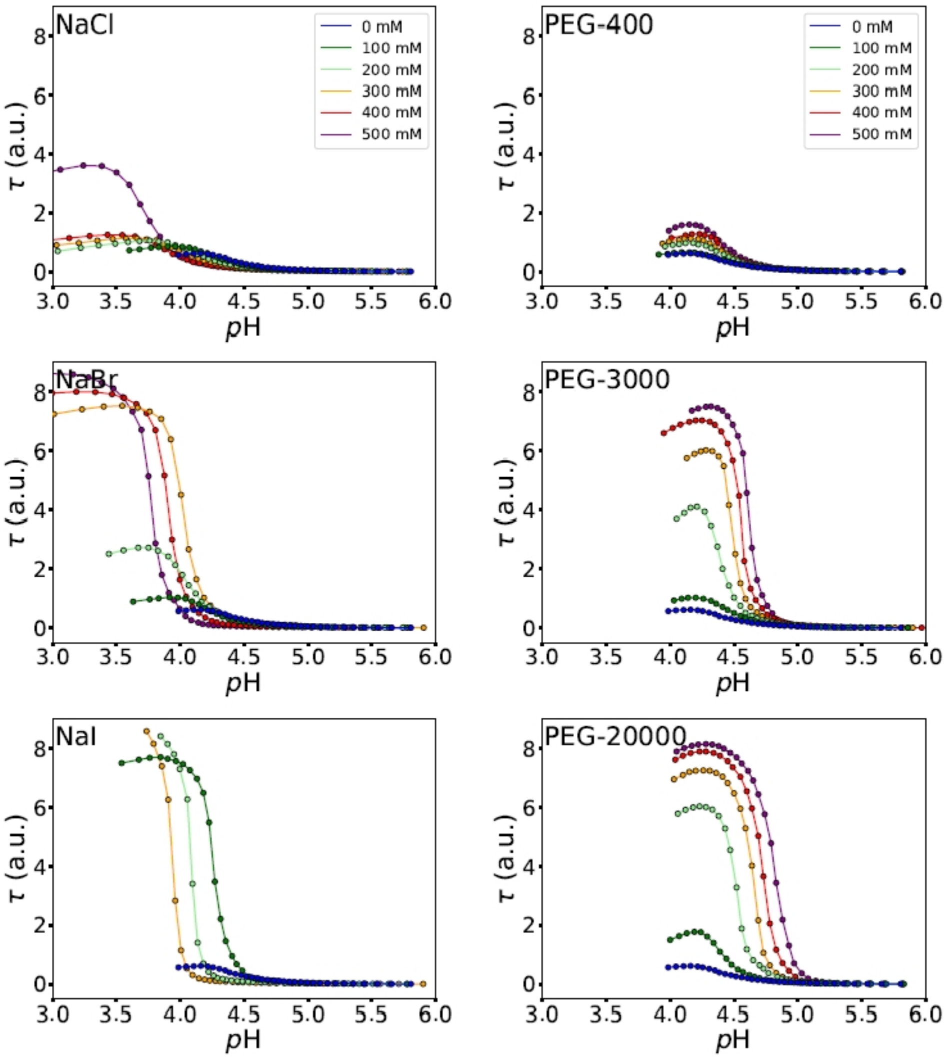

Figure S2. Turbidimetric titration curves for solutions containing 100 μM BSA and 4000 μM NaPSS (r = 40) in acetate buffer (initial pH = 5.8, ionic strength 0.1 M) in the absence and presence of various salts (NaCl, NaBr, NaI; left) and polyethylene glycols (PEG-400, PEG-3000, PEG-20000; right). Concentrations of co-solute were 0, 100, 200, 300, 400, and 500 mM. The pH was changed by titrating the solution with 0.2 M HCl solution. Data correspond to 25 °C.

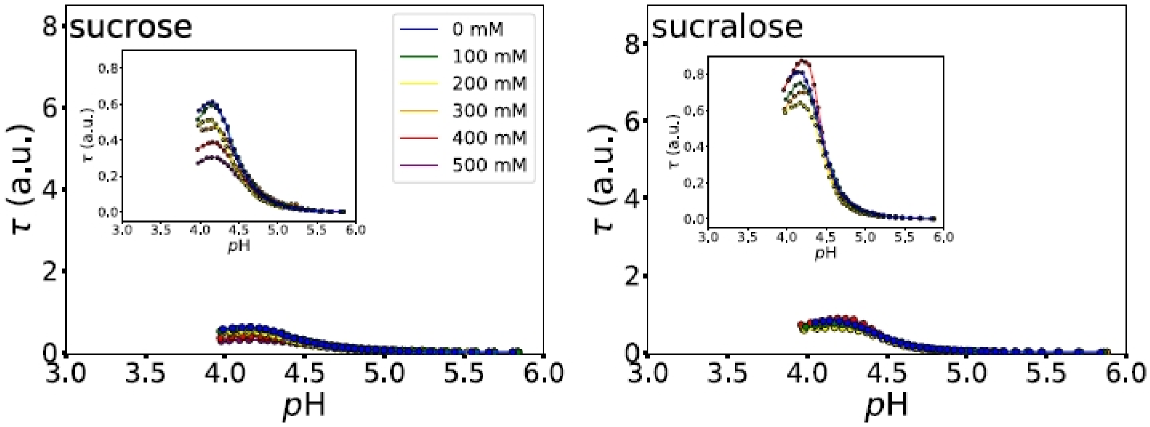

Figure S3. Same as in Figure S2 but for solutions with sucrose (left) and sucralose (right).

Section S3. BSA/NaPSS complexation is accompanied by BSA aggregation

The increase in turbidity upon acidification of the BSA/NaPSS solutions with or without co-solutes (Figures S2 and S3) is a consequence of two processes: (i) protein-PE complexation and (ii) protein-protein aggregation. Both complexation and aggregation are in principle modulated by co-solutes. Fiure S4 gives a comparisson of turbidimetric titration curves of BSA-NaI and BSA/NaPSS-NaI solutions for three salt concentrations (100, 200, and 300 mM). We can see that the presence of NaI promotes the aggregation of BSA molecules because the salt ions screen the repulsive electrostatic interactions between BSA molecules. The effect is most obvious for BSA and BSA/NaPSS in the presence of 300 mM NaI, where the initial increase in turbidity (4.2 < pH < 5.0) is mainly related to the formation of BSA-NaPSS complexes, while at pH < 4.2 the increase in τ is largely also due to BSA aggregation.

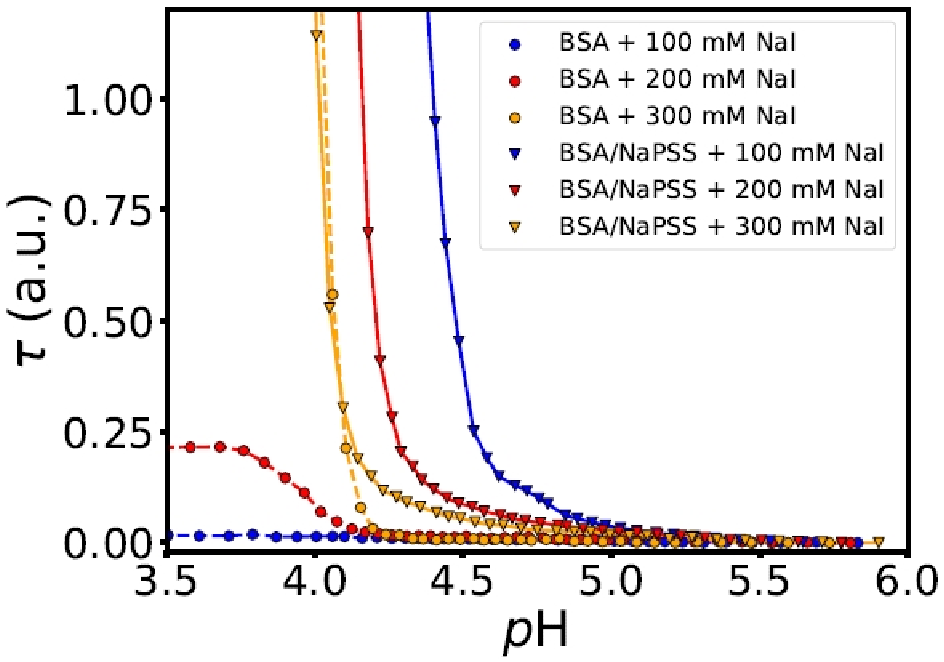

Figure S4. Turbidimetric titration curves for solutions containing 100 μM BSA and NaI (circles) or 100 μM BSA, 4000 μM NaPSS (r = 40) and NaI (triangles) in acetate buffer (initial pH = 5.8, ionic strength 0.1 M). The concentrations of NaI were 100, 200, or 300 mM. Data correspond to 25 °C.

Photographic images of the BSA/NaPSS (r = 40) systems without or with 100 mM NaI at $p\mathrm{H}_{\mathrm{opt}}$ taken with an optical microscope are shown in Figure S5. The presence of 100 mM NaI stimulates precipitation of protein-PE complexes (right) – especially at pH values below the isoionic point of BSA – leading to an increase in turbidity of the solution compared to the salt-free system (left). Since this is connected with the BSA/NaPSS complexation as well as BSA aggregation (see Figure S4), formed complexes as a result of BSA-NaI interaction, have a higher content of protein molecules if compared to the salt-free system.

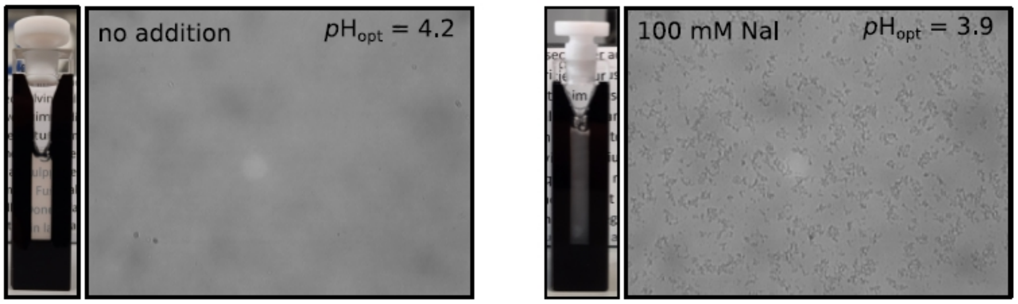

Figure S5. Optical microscopy images at $p\mathrm{H}_{\mathrm{opt}}$ of salt-free BSA/NaPSS solution (left) and for BSA/NaPSS solution with 100 mM NaI (right). In both cases r = 40 and T = 25 °C.

Section S4. Interaction of PEG with hydrophobic surface of BSA is negligible

The fluorescence emission spectra of BSA-PEG solutions (PEG-3000 and PEG-20000) in acetate buffers (pH = 4.2 and pH = 5.8) are for different PEG concentrations shown in Figure S6 (excitation wavelength 280 nm). The changes in the spectra with respect to pH, molecular weight of PEG and PEG concentration are marginal. The corresponding results of the fluorescence quenching analysis (Stern-Volmer plots) are shown in Figure S7. We see that the quenching is minimal in the studied concentration range of PEG (see also Table 1 in the main article), indicating negligible BSA-PEG interactions.

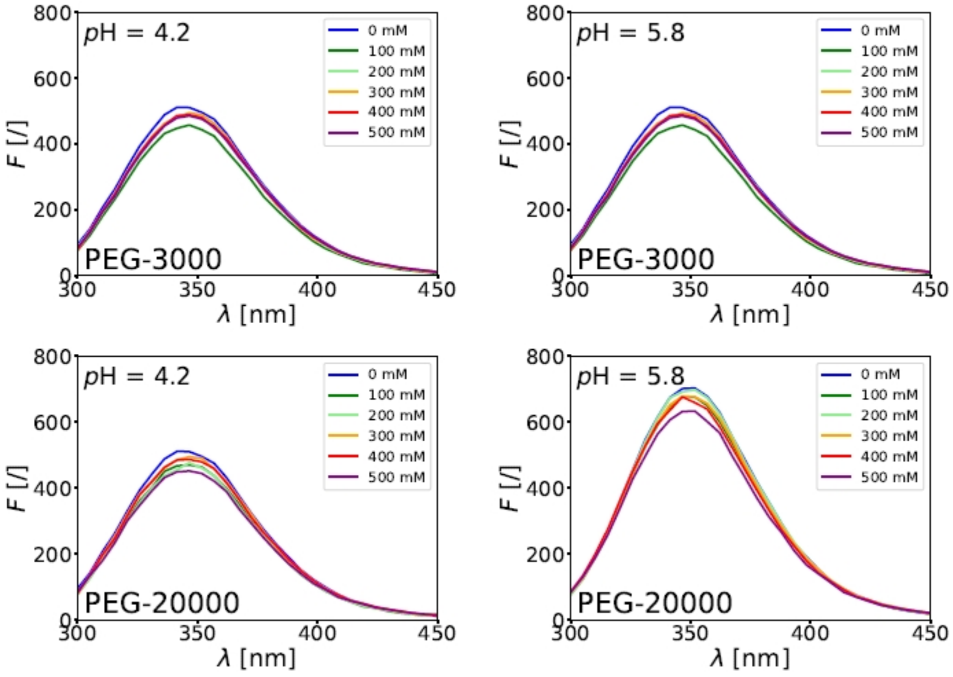

Figure S6. Emission spectra of 0.5 μM BSA solutions with added PEG-3000 and PEG-20000 for different PEG concentrations. Solutions were prepared in acetate buffer with pH = 5.8 and 4.2(I = 0.1 M). Data correspond to 25 °C.

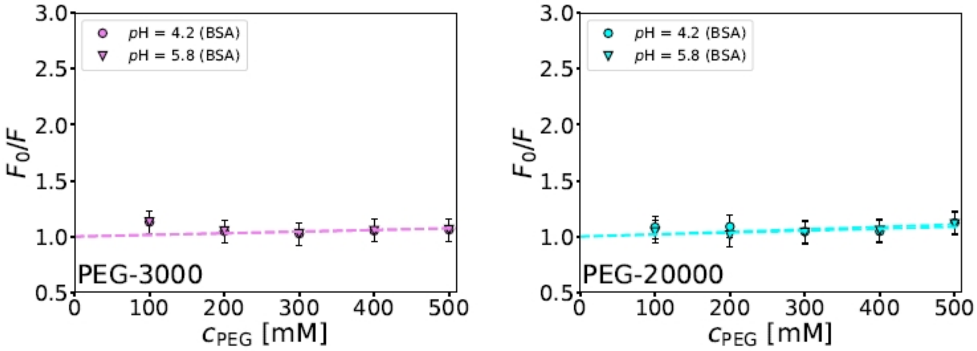

Figure S7. Stern-Volmer plots for PEG-3000 (left) and PEG-20000 (right). Solutions were prepared in acetate buffers with pH = 5.8 and 4.2 (I = 0.1 M). Data correspond to 25 °C.

Section S5. Interaction of sugars with hydrophobic surface of BSA

The fluorescence emission spectra of BSA sugar solution (sucrose and sucralose) in acetate buffer (pH = 4.2 and pH = 5.8) are shown for different sugar concentrations in Figure S8 (excitation wavelength 280 nm). It is evident that the presence of sucrose does not drastically affect the emission spectrum of BSA above or below the isoionic point of BSA, however, the presence of sucralose drastically changes the fluorescence emission. Changes are more pronounced at pH above the isoionic point. The change in the emission

spectrum is related to changes in the molecular environment of the fluorophores, which may be a consequence of either direct (co-solute)-protein interactions or structural changes in the protein (induced by the co-solute).

Since all of our solutions were prepared in high ionic strength acetate buffer, which absorbs strongly at shorter wavelengths (4 220 nm), direct CD spectra measurements could not be made. However, considering that the secondary structure of BSA is predominantly composed of alpha helices (approx. 67%), the extent of potential conformational changes induced by co-solutes can be estimated at two characteristic wavelengths, 208 and 222 nm [1]. To estimate the extent of possible conformational changes, we measured the mean residue ellipticity (MRE), [θ], at 222 nm for 4.5 μM BSA in the presence of various sugar concentrations (Table S1). MREs were determined as the average of two measurements, with the maximum error estimated to be ±1 unit. The resulting MREs at 222 nm are within experimental error regardless of sugar, suggesting that the presence of sugar (in the entire concentration interval) does not affect the secondary structure of BSA. The fluorescence quenching data can therefore be considered as a result of direct BSA-sugar interactions.

Table S1. Mean residue ellipticity at 222 nm, [θ] (10^3 deg cm^2 dmol^{-1}), for BSA solutions with added sucrose or sucralose as a function of sugar concentration, c. Solutions were prepared in acetate buffer (pH = 4.2, I = 0.1 M). Data apply for T = 25 °C. The error in [θ] was estimated to be ±1.

c [mM]	[θ] (sucrose)	[θ] (sucralose)
0	−19.3	−19.3
100	−18.8	−19.6
200	−19.8	−20.1
300	−19.7	−20.2
400	−19.7	−19.1

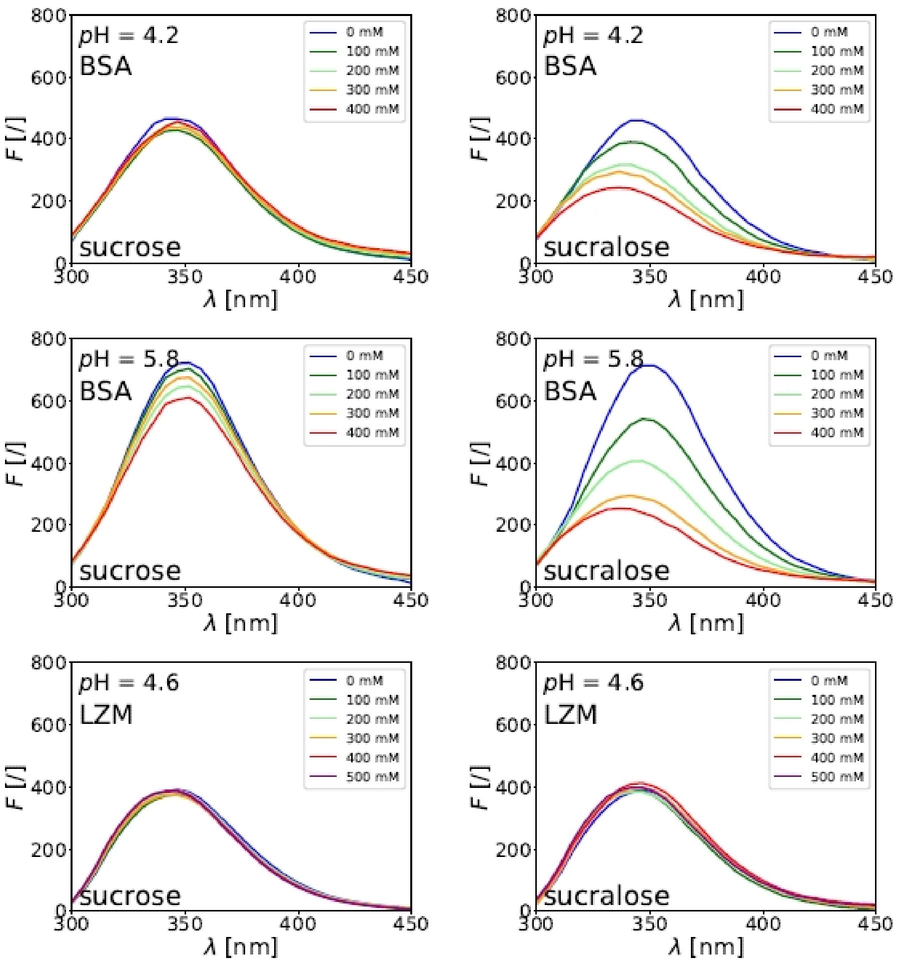

Figure S8. Emission spectra of 0.5 *m*M BSA and 2 *m*M lysozyme (LZM) with added sugar (sucrose and sucralose) for different sugar concentrations. Solutions were prepared in acetate buffer with ionic strength of I = 0.1 M at pH = 5.8 and 4.2 for BSA and pH = 4.6 for LZM. Data correspond to 25 °C.

Section S6. Local distribution of sugar and water molecules around the protein surface

As shown by the fluorescence measurements, sucralose molecules have a stronger tendency to hydrophobic pockets of BSA compared to sucrose (see Section SS5 in this file). Moreover, quenching analysis (Stern-Volmer plots; Figure 7b) indicates that the affinity of sucralose toward lipophilic pockets is higher at *p*H = 5.8 than at *p*H = 4.2. Considering that changes in the *p*H value of the medium affect the protonation states of acidic/basic amino acid residues at the protein surface, it is reasonable to conclude that sucralose also exhibits an affinity for charged residues [2]. Since glutamic acid (GLU) residues have a $pK_a \approx (4.2 - 4.4)$, the protonation state of GLU could be responsible for the differences seen in Figure 7b. To complement these findings, we used molecular dynamics (MD) simulations to calculate the average number of nearest neighbour sugar oxygen atoms (similar to Figure 8 in the main article) from the BSA surface (including hydrogen atoms). Histograms for gluatmic acid and tryptophan (TRP) at both *p*H values are shown in Figure S9. The sampling was similar to that used for the histogram in the main text (Figure 8), but we included only GLU and TRP residues. From Figure S9, it can be seen that more sucralose molecules cluster around GLU residues at *p*H = 5.8. At this *p*H, the GLU residues are deporotonated and the carboxylate groups (COO^-) act as stronger HB acceptors, while at *p*H = 4.2, only some of these groups are in the ionic form. MD simulations show no significant accumulation of sugar molecules around TRP, which are found in the hydrophobic pocket of the protein. BSA has only two TRP while it has 59 GLU. Although histograms are given per number of a given amino acid, the statistics of accumulation over a 25 ns simulation are much weaker for TRP than for GLU. In addition, hydrogen bonding and Coulomb interactions are energetically more favourable than van der Waals interactions, so the greater tendency of polar sugar molecules to polar/charged amino acid residues is a consequence of the force field used. The results of fluorimetry and MD simulations should therefore be considered complementary rather than mutually exclusive.

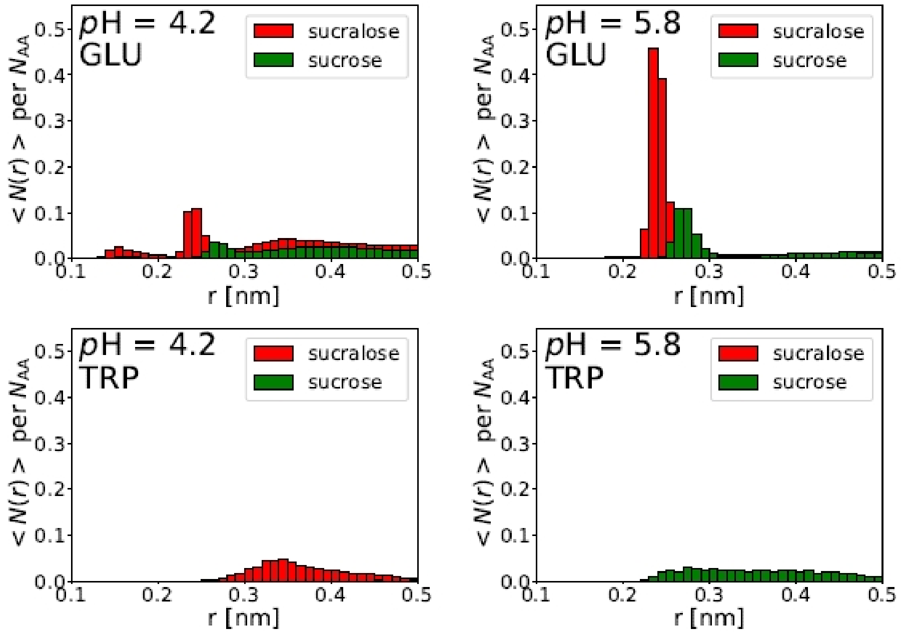

Figure S9. Average number of closest sugar oxygen atoms in the vicinity of GLU (top) and TRP (bottom), grouped into 0.01 nm wide bins, and normalized per number of amino acid residues

(closestprotein atom including hydrogen atoms was considered). Data are for 25 ns MD simulations in SPC/E water at 25 °C for *p*H = 4.2 (left) and 5.8 (right).

To highlight the local distribution of water (and sugar) molecules around the proteinsurface we calculated the time-averaged normalized ratio $G_{ow}(r)$: [2,3]

$$G_{OW}(r) = \frac{1}{N_{norm}}\left(\frac{n_{OW}}{n_{OW} + n_{OS}}\right) \quad (1)$$

where n_{ow} and n_{os} represent the local numbers of water oxygen atoms and all sugar hydroxyl oxygens, respectively. The normalization constant, N_{norm}, is calculated as the average ratio $n_{ow}/(n_{ow} + n_{os})$ between 1.5 and 3.0 nm from the protein's surface. At those distances the interactions between the amino acid residues and sugar (water) molecules canbe neglected, meaning that sugar/water molecules represent the bulk phase. If $G_{ow}(r) > 1$ this indicates that the protein surface is preferentially hydrated. In contrast, if $G_{ow}(r) < 1$ water is displaced and the sugar is preferentially bound to the protein surface. Figure S10 shows $G_{ow}(r)$ for BSA-sucrose and BSA-sucralose solutions at *p*H = 4.2 (left) and 5.8 (right). The distribution for sucralose indicates that this sugar is indeed preferentially bound to the BSA surface ($G_{ow} \ll 1$ at small *r*). The two minima at around 0.160 and 0.240 nm correspond roughly to the peaks in Figure 8 in the main text. The G_{ow} for sucrose suggests that this sugar interacts only weakly with the protein surface (a small minimum is observed at approx. 0.240 nm), with the interaction of sucrose being more water-mediated if compared to sucralose.

The consequence of a higher propensity of sucralose to the protein surface (in addition to water displacement) explains the hindered onset of BSA/NaPSS complexation (pH$_c$) upon acidification (Figure 6a in the main text) since tightly bound sucralose molecules to the BSA surface prevent the contact between BSA and NaPSS.

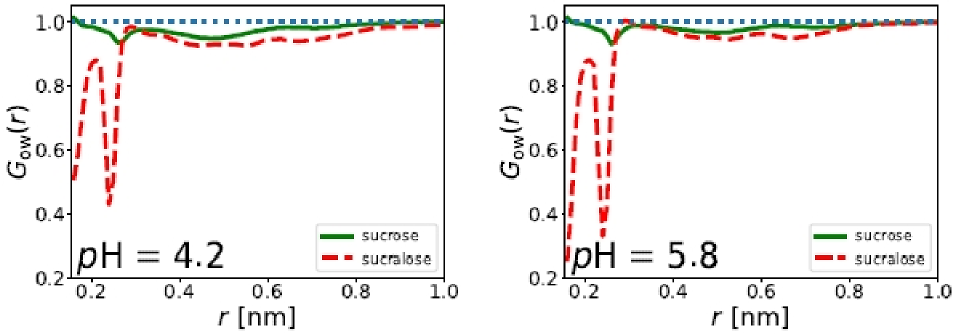

Figure S10. Time-averaged normalized fraction of water oxygen atoms, $G_{OW}(r)$, as a function of thedistance to the closest BSA atom, *r* (*cf*. Equation (1), for BSA-sucrose (green) and BSA-sucralose (red) solutions at *p*H = 4.2 (left) and 5.8 (right). Protonation states of BSA at given *p*H were estimated by PROPKA [4]. Data are for 25 ns MD simulations in SPC/E water at 25 °C.

References

1. Wei, Y.; Thyparambil, A.A.; Latour, R.A. Protein helical structure determination using CD spectroscopy for solutions with strong background absorbance from 190 to 230nm. *Biochim. Biophys. Acta (BBA) - Proteins Proteom.* **2014**, *1844*, 2331–2337.
2. Simončič, M.; Lukšič, M. Mechanistic differences in the effects of sucrose and sucralose on the phase stability of lysozyme solutions. *J. Mol. Liq.* **2021**, *326*, 115245.
3. Lerbret, A.; Bordat, P.; Affouard, F.; Hédoux, A.; Guinet, Y.; Descamps, M. How Do Trehalose, Maltose, and Sucrose Influence Some Structural and Dynamical Properties of Lysozyme? Insight from Molecular Dynamics Simulations. *J. Phys. Chem. B* **2007**, *111*, 9410–9420.
4. Olsson, M.H.; Søndergaard, C.R.; Rostkowski, M.; Jensen, J.H. PROPKA3: consistent treatment of internal and surface residues in empirical pK_a predictions. *J. Chem. Theory Comput.* **2011**, *7*, 525–537.
